# Designing novel peptides with amyloid-*β* binding and clearance potential using BiLSTM and molecular dynamics

**DOI:** 10.3389/frai.2025.1709505

**Published:** 2025-11-19

**Authors:** Vinod Kumar Yata, Om Pritam Das, Jarmani Dansana, Abhishikta Gadtya, Biswa Ranjan Meher, Sarad Pawar Naik Bukke, Narasaiah Kolliputi

**Affiliations:** 1 Department of Biotechnology, School of Allied and Healthcare Sciences, Malla Reddy University, Hyderabad, Telangana, India; 2 Department of Molecular Biology, Central University of Andhra Pradesh, Ananthapuramu, Andhra Pradesh, India; 3 Computational Biology and Bioinformatics Laboratory, PG Department of Botany, Berhampur University, Berhampur, Odisha, India; 4 Department of Pharmaceutics and Pharmaceutical Technology, Kampala International University, Western Campus, Ishaka, Uganda; 5 Division of Allergy and Immunology, Department of Internal Medicine, USF Morsani College of Medicine, Tampa, FL, United States

**Keywords:** deep learning, BiLSTM, peptide design, amyloid-*β*, gene ontology annotation, molecular dynamics

## Abstract

Generative artificial intelligence is transforming de novo biomolecular design, yet developing models that reliably generate functional, target-specific peptides remains a significant challenge. Here, we introduce and validate a novel two-stage Bidirectional Long Short-Term Memory (BiLSTM) framework for the generative design of short, functional peptides. Our AI pipeline is trained on full-length proteins annotated with specific Gene Ontology (GO) terms related to amyloid-*β* (Aβ) interaction and is fine-tuned on experimentally validated peptide fragments to capture local functional motifs within a global protein context. As a proof-of-concept, we applied this framework to generate peptides targeting Aβ42, a key pathological agent in Alzheimer’s disease. From 1,000 AI-generated sequences, 25 candidates were shortlisted using biophysical filters (GRAVY, instability index, Shannon entropy), and 11 were prioritized via sequence similarity analysis, designated as AI-Designed Novel Peptides (ADNP1-ADNP11). Structural modeling (AlphaFold2) and docking (pyDockWEB) against Aβ42 identified ADNP7 as the top candidate, exhibiting a highly favorable docking score (−63.33 kcal/mol), with interactions localized to Aβ’s aggregation-prone regions. All-atom molecular dynamics simulations (20 ns) confirmed complex stability, and MM/PBSA analysis yielded a strong binding free energy (−50.6 kcal/mol), driven primarily by hydrophobic and aromatic interactions involving PHE12 and TRP50 in ADNP7. This work demonstrates that our fine-tuned BiLSTM architecture can successfully generate novel, stable peptide sequences with high predicted binding affinity for a therapeutically relevant target. While the training data included proteins associated with Aβ clearance (GO:0097242), only binding interactions were computationally validated; clearance potential remains a hypothesis for future experimental testing. This study establishes a generalizable, AI-driven pipeline for functional peptide design, with broad applicability across therapeutic discovery and synthetic biology.

## 1 Introduction

Alzheimer’s disease (AD) is a progressive neurodegenerative disorder associated with the accumulation of amyloid-*β* (Aβ) aggregates (Finder and Glockshuber, 2007; Scheltens et al., 2021). Among its isoforms, Aβ42 is particularly prone to aggregation and neurotoxicity, driving synaptic dysfunction, oxidative stress, and neuroinflammation that contribute to cognitive decline (Younkin, 1998; Mayeux et al., 2003; Kuperstein et al., 2010). Despite extensive efforts with small molecules, monoclonal antibodies, and enzyme-based clearance strategies, clinical outcomes remain limited due to poor blood–brain barrier (BBB) penetration and insufficient targeting of oligomeric or fibrillar Aβ species (Levites et al., 2006; Weggen et al., 2007; Miners et al., 2008; Citron, 2010; Nie et al., 2011). Peptide-based therapeutics have emerged as promising alternatives, offering tunable specificity, reduced immunogenicity, and comparatively favorable BBB permeability relative to larger biologics (Funke and Willbold, 2012; Goyal et al., 2017). Their modularity allows for rational design and fine-tuning of affinity and selectivity (McGregor, 2008; Kaspar and Reichert, 2013). However, the development of multifunctional peptides that combine strong Aβ binding with stability and clearance potential remains challenging, largely due to the conformational heterogeneity of Aβ assemblies (Tomaselli et al., 2006; Fändrich et al., 2009). Previous efforts have explored peptidomimetics, binder–blocker sequences targeting motifs such as KLVFF, and mimetic immunotherapies, but these approaches often rely on predefined motifs or rational engineering, limiting the discovery of truly novel candidates (Lowe et al., 2001; Chafekar et al., 2007; Morgan, 2011; Goyal et al., 2017; França et al., 2024).

Recent advances in artificial intelligence (AI) provide new opportunities to address these challenges by incorporating large biological datasets and machine learning/ deep learning algorithms (Fabrizio et al., 2021). LSTM-based models have been applied to identify bioactive motifs, CNN-BiLSTM hybrids to predict multifunctional peptide activities, and generative frameworks to design antiviral peptides (Fabrizio et al., 2021; Xiao et al., 2021; Li et al., 2022). Docking and molecular dynamics (MD) simulations have further elucidated Aβ interaction mechanisms (Urbanc et al., 2004; Mandal et al., 2006; Zhang et al., 2021). Yet, most approaches remain fragmented, focusing either on predictive modeling or structural analysis without fully integrating AI-driven peptide generation with physics-based validation.

To overcome these limitations, we developed a two-stage Bidirectional LSTM (BiLSTM) framework trained on proteins annotated for Aβ binding and clearance (GO:0001540 for Aβ binding and GO:0097242 for Aβ clearance), followed by fine-tuning with short peptide fragments to capture therapeutically relevant motifs. From 1,000 generated sequences, we applied multi-level filtering incorporating physicochemical properties, sequence diversity, structural prediction, docking, and MD simulations. Eleven candidates (ADNP1-ADNP11) were identified, with ADNP7 showing the most favorable stability and binding profile against A*β*42. Collectively, this study introduces an AI-guided pipeline that integrates generative deep learning with structural and energetic validation, offering prioritized peptide leads for AD and a broadly applicable strategy for therapeutic peptide discovery.

## 2 Methods

### 2.1 Study design and architecture

This study introduces a novel, end-to-end computational framework for the AI-driven generative design of functional peptides, demonstrated through the targeted generation of peptides predicted to bind amyloid-*β* (Aβ42). The architecture is centered on a two-stage Bidirectional Long Short-Term Memory (BiLSTM) (Berglund et al., 2015) generative model, explicitly designed to bridge the gap between high-level biological function (encoded via Gene Ontology annotations) and local, therapeutically relevant peptide motifs. The pipeline is modular, reproducible, and generalizable; while applied here to Aβ, it can be readily adapted to other protein targets by substituting the training dataset and validation structure. At its core, the BiLSTM generative model (Berglund et al., 2015) learns to predict peptide sequences by modeling the conditional probability distribution of amino acid residues in a sequence. Formally, the probability of generating a peptide sequence $a=(a_{1},a_{2},\ldots ,a_{T})$ of length, T is factorized as the product of conditional probabilities:


$$P(a)=\prod_{t=1}^{T}P(a_{t}|,,,;a_{1}|,,,;a_{2}|,,,;\ldots |,,,;a_{t-1}|,,,;\theta )$$

where, $a_{t}$ represents the amino acid at position- t , and, θ denotes the model parameters learned during training. This autoregressive modeling enables the BiLSTM to capture complex sequence dependencies in both forward and backward directions, crucial for generating biologically meaningful peptides.

**Figure 1:**
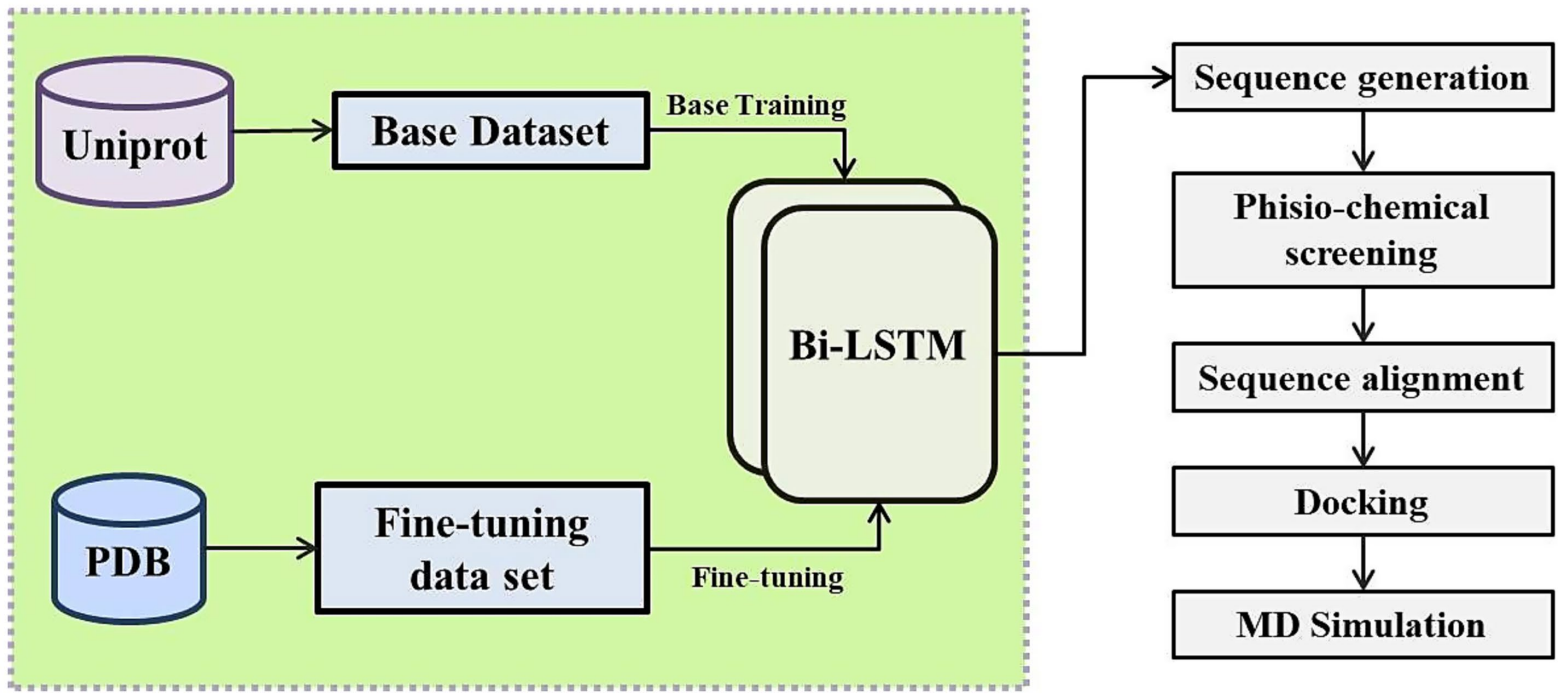
Schematic overview of the deep learning-based pipeline for the generation, screening, and validation of novel peptides amyloid-beta binding and clearance potential.

The overall workflow consists of four tightly integrated phases: (1) generative modeling via the fine-tuned BiLSTM network trained initially on full-length functional proteins and subsequently refined on short peptide fragments; (2) multi-parameter biophysical screening to prioritize stable, non-repetitive, and novel sequences; (3) structural modeling and rigid-body docking to assess binding potential against Aβ42; and (4) all-atom molecular dynamics simulations coupled with MM/PBSA energetic profiling to validate complex stability and interaction mechanisms. This design ensures that AI-generated outputs are not only novel and diverse but also rigorously validated across sequence, structure, and dynamics levels prior to experimental testing (see Figure 1).

The core innovation lies in the two-stage training strategy: initial exposure to broad functional contexts (GO:0001540 for Aβ binding and GO:0097242 for Aβ clearance) (Ashburner et al., 2000) followed by fine-tuning on compact, experimentally resolved peptide fragments. This approach enables the model to generate short sequences (<100 residues) that retain essential functional signatures without relying on direct templating. All generated candidates are designated as “AI-Designed Novel Peptides (ADNP)” to accurately reflect their AI-driven origin. The pipeline’s emphasis on iterative computational validation from sequence generation to dynamic stability ensures robustness and provides a template for AI-driven peptide discovery across various therapeutic domains.

### 2.2 Data obtaining and preparation

The dataset for model development was manually curated to support the generation of peptides targeting amyloid-beta pathology. It comprises experimentally validated protein and peptide sequences with known amyloid-beta binding, uptake, or degradation activity, and is organized into two distinct subsets, as summarized in Table 1: The Background (Base) Set includes 7 full-length protein sequences retrieved from the UniProt database (The UniProt Consortium, 2021). These proteins (sequence lengths ranging from 230 to 1,019 residues) were selected for their relevance in amyloid-beta clearance pathways and used to pretrain the model on general sequence patterns and motif structures. The Finetuning (Sample) Set consists of short 6 peptide chains (≤100 residues) derived from crystallographic structures in the Protein Data Bank (PDB) (Berman et al., 2000). These peptides represent spatially resolved regions directly implicated in amyloid-beta interaction and were used to fine-tune the model toward generating bioactive peptides with therapeutic potential.

**Table 1:** Summary of protein sequences used for model training and fine-tuning, including UniProt/PDB IDs, sequence lengths, and literature references.

Background (Base) Set			
Uniprot id	Protein Name	Sequence Length	Ref.
P10909	Clusterin	449	Narayan et al. (2012) and Yeh et al. (2016)
P11835	Integrin beta-2	771	Jeon et al. (2008) and Choucair-Jaafar et al. (2011)
P16671	Platelet glycoprotein 4	472	Shimizu et al. (2008) and Zhao et al. (2018)
P30204	Macrophage scavenger receptor types I and II	458	Husemann et al. (2001)
P35559	Insulin-degrading enzyme	1,019	Llovera et al. (2008), Shimizu et al. (2008), and Vekrellis et al. (2000)
P35951	Low-density lipoprotein receptor	862	Kim et al. (2009) and Basak et al. (2012)
Q9NZC2	Triggering receptor expressed on myeloid cells 2	230	Yeh et al. (2016), Zhao et al. (2018), and McQuade et al. (2020)

Finetuning (Sample) Set			
PDB Id	Protein Name	Sequence Length	Ref.
2FYL	alpha2-macroglobulin receptor-associated protein Chain 1	81	Basak et al. (2012)
	alpha2-macroglobulin receptor-associated protein Chain 2	82	
6V7M	Apolipoprotein E Chain 1	100	Cho et al. (2001) and Hopkins et al. (2011)
	Apolipoprotein E Chain 2	83	
2KNX	Low-density lipoprotein receptor-related protein 1	50	Bell et al. (2009), Kanekiyo et al. (2012, 2013), and Zhao et al. (2015)
2KNY	Prolow-density lipoprotein receptor-related protein 1	80	

All sequences were derived from peer-reviewed studies and cross-validated for biological relevance. No synthetic or computationally augmented sequences were introduced.

Encoding and Sequence Windowing.

Each amino acid sequence was processed using a standard integer encoding scheme over the 20 canonical amino acids:


A={A,C,D,E,F,G,H,I,K,L,M,N,P,Q,R,S,T,V,W,Y}

Given sequence $\;S=(s_{1},s_{2},\ldots ,s_{L})$ , the encoded representation is defined as:


$$\hat{S}=(f(s_{1}),f(s_{2}),\ldots ,f(s_{L})),\text{wheref}:A\to \{0,1,\ldots ,19\}$$

To construct training examples for the generative model, a sliding window of length 35 was applied to each encoded sequence. For each window, the first 34 residues served as the input, and the 35th residue was used as the target token. Formally, the training pair at position *i* is:


$$X_{i}=({\hat{s}}_{i},{\hat{s}}_{i+1},\ldots ,{\hat{s}}_{i+33}),y_{i}={\hat{s}}_{i+34}$$

This framing defines a next-token prediction task, enabling the model to learn the conditional probability of the next amino acid given its context:


$$P(x_{t}|,,,x_{1}|,,,x_{2}|,,,\ldots |,,,x_{t-1})$$

All sequences were truncated or padded to ensure consistency in length where necessary, but no structural or contextual augmentation was performed. This ensured that all training data remained biologically grounded, reflective of experimentally verified interactions with amyloid-beta.

### 2.3 Model development and training

The generative model was constructed using a bidirectional Long Short-Term Memory (BiLSTM) neural network architecture, chosen for its ability to capture long-range contextual dependencies within peptide sequences (Berglund et al., 2015). Unlike unidirectional models, BiLSTMs process input in both forward and reverse directions, making them well-suited to identifying biologically relevant sequence motifs in both N-terminal and C-terminal contexts. Peptide sequences were first integer-encoded using a fixed vocabulary of the 20 standard amino acids. A sliding window of length 35 was applied to each sequence, generating overlapping subsequences in the format $\;(x_{1},x_{2},\ldots ,x_{34})\to x_{35}$ , where the first 34 residues served as input and the 35th residue was used as the target label. The model was thus trained to learn the conditional probability distribution over amino acids given a preceding context window:


$$P(x_{t}|,,,x_{1}|,,,x_{2}|,,,\ldots |,,,x_{t-1})$$

At each position t , the BiLSTM model produced forward and backward hidden states defined as:


$${\vec{h}}_{t}=\mathrm{LST}M_{f}(x_{t},{\vec{h}}_{t-1}),{\overset{´}{h}}_{t}=\mathrm{LST}M_{b}(x_{t},{\overset{´}{h}}_{t+1})$$

The final representation at position t was obtained by concatenating these directional states:


$$h_{t}=[{\vec{h}}_{t};{\overset{´}{h}}_{t}]$$

This hidden representation was passed through two fully connected dense layers with ReLU activations, followed by a final softmax layer to output the predicted probability distribution over the 20 amino acid classes:


$${\hat{y}}_{t}=\text{Softmax}(Wh_{t}+b)$$

Where W and b are the learnable weights and biases of the output layer. Model training was guided by the categorical cross-entropy loss function:


$$L=-\sum_{t=1}^{T}y_{t}\log({\hat{y}}_{t})$$

The full architecture consisted of two stacked BiLSTM layers with 256 units each, followed by two dense layers. Dropout regularization ( dropout rate=0.2 ) was applied after each LSTM layer to mitigate overfitting. The model was trained using the Adam optimizer (learning rate = 0.001) for 150 epochs in the base phase and 45 epochs in the fine-tuning phase, with batch sizes of 30 and 62, respectively.

Training was carried out in two distinct phases. During the base phase, the model was trained on long, functionally annotated protein sequences (Table 1), enabling it to learn generalizable sequence grammar related to amyloid-beta interaction. In the second phase, the model was fine-tuned using a curated dataset of shorter peptides (<100 residues), steering its output distribution toward compact, therapeutically relevant sequences. The model was optimized using the Adam optimizer with a learning rate of $1\times 10^{-3}$ , a batch size of 64 for generalization, and early stopping (patience = 10) based on validation loss. Although validation split was not explicitly implemented in code due to the limited sample size, instead stability and convergence of the model were monitored by tracking training loss across epochs and performing repeat runs to assess reproducibility. This two-phase training paradigm allowed the model to first learn the global syntax and compositional structure of bioactive peptides and then specialize in generating novel, functionally coherent, and sequence-stable peptides suitable for downstream screening.

### 2.4 Peptide generation and physicochemical shortlisting

Following fine-tuning, the trained BiLSTM model was utilized to generate novel peptide sequences through an autoregressive sampling approach. Starting with randomly constructed seed sequences derived from the training dataset, the model predicted the next amino acid token iteratively. At each step t , the model used a fixed-length context window of 35 residues and predicted the most probable next amino acid $x_{t+1}$ based on the preceding sequence $\;x_{t-34:t}$ :


$$P(x_{t+1}|,,,x_{t-34}|,,,x_{t-33}|,,,\ldots |,,,x_{t})=\text{softmax}(f_{\text{BiLSTM}}(x_{t-34:t}))$$

Here, $f_{\text{BiLSTM}}$ denotes the trained network and the output is sampled via greedy decoding (argmax). This process was repeated until a complete peptide of length, L∈[80,100] residues was generated. A total of 1,000 such sequences were synthesized.

To systematically reduce the sequence space, a two-stage physicochemical screening pipeline was employed. In Stage 1, the generated sequences were partitioned into five batches of 200 each. For every peptide, the following three metrics were computed:

Shannon entropy H , measuring amino acid diversity within a sequence s of length n :


$$H(s)=-\sum_{a\in A}p(a)\log_{2}p(a)$$

where A is the set of standard amino acids and p(a) is the empirical frequency of residue a in the sequence (Shannon, 1948).

GRAVY score G , calculated as the mean hydropathy index over all residues:


$$G(s)=\frac{1}{n}\sum_{i=1}^{n}h(s_{i})$$

where $h(s_{i})$ is the Kyte-Doolittle hydropathy value of residue $s_{i}$ (Kyte and Doolittle, 1982).

Instability Index I , which estimates *in vitro* stability based on dipeptide composition. While the precise formula involves 400 pairwise weights $\delta (s_{i},s_{i+1})$ , it is conceptually represented as:


$$I(s)=\frac{10}{n}\sum_{i=1}^{n-1}\delta (s_{i},s_{i+1})$$

Higher values of I indicate greater instability; sequences with I>40 are generally considered unstable (Guruprasad et al., 1990).

Within each batch, sequences were ranked to prioritize high Shannon entropy, moderate-to-low GRAVY, and low instability index. The top 10 sequences per batch were selected, yielding 50 candidates for further evaluation.

In Stage 2, these shortlisted peptides were compared to those in the fine-tuning dataset to ensure alignment with physicochemical properties of experimentally validated Aβ-interacting peptides. For each generated sequence p and reference peptide r , the Euclidean distance in the 2D feature space of GRAVY and instability was computed as:


$$d(p,r)=\sqrt{{\left(G_{p}-G_{r}\right)}^{2}+{\left(I_{p}-I_{r}\right)}^{2}}$$

Each batch of 10 sequences was compared to the centroid of the fine-tuning reference distribution, and the five closest sequences per batch were retained, resulting in 25 final candidate peptides. This two-stage process ensured that the selected peptides possessed sequence diversity, biophysical stability, and feature similarity to known functional peptides, while maintaining novelty.

### 2.5 Sequence similarity analysis

To assess the novelty and potential functional relevance of the shortlisted peptides, sequence similarity analysis was performed using Clustal Omega, a widely accepted tool for multiple sequence alignment (Sievers and Higgins, 2014). This step ensured that the generated sequences shared meaningful similarity with known amyloid-beta-binding and degrading peptides, while still maintaining a level of novelty indicative of *de novo* design. The 25 shortlisted sequences were aligned against the six fine-tuning peptide chains [derived from PDB entries Clusterin (2FYL), Heat shock protein HSP 90-beta (6V7M), Metalloprotease (2KNX), and Metalloprotease domain-containing protein 3 (2KNY) using Clustal Omega with default parameters]. The alignment output provided pairwise percentage identity scores for each generated peptide against the reference chains. These scores were analyzed to identify which sequences shared the highest similarity to functionally validated peptides, serving as a proxy for potential biological relevance.

### 2.6 3D modelling and docking analysis

To assess the structural plausibility and amyloid-beta (Aβ) binding potential of the shortlisted peptides, 3D structural modeling followed by protein–protein docking was performed. The 11 peptides selected after biophysical screening and sequence similarity analysis were named as Amyloid-Degrading Novel Peptide (ADNP1–ADNP11) and were structurally modeled using the AlphaFold server (Jumper et al., 2021). Structural visualization and verification were carried out using Jmol to ensure correct folding, absence of steric clashes, and suitability for docking (Herráez, 2006). Protein–protein docking was performed using pyDockWEB, a rigid-body docking server that incorporates electrostatics, desolvation, and van der Waals scoring (Jiménez-García et al., 2013). Each modeled peptide structure was docked against the Aβ42 monomer structure retrieved from the Protein Data Bank (PDB ID: 1IYT) (Crescenzi et al., 2002). The docking protocol evaluated multiple energy-based scoring components, including electrostatic energy, desolvation energy, van der Waals energy, and total binding energy. ADNP peptides demonstrating more negative total docking scores were interpreted to have higher binding affinities toward Aβ. Particular emphasis was placed on peptides exhibiting favorable van der Waals and desolvation scores, as these indicate better surface complementarity and solvent compatibility, both of which are crucial for stable and specific protein–protein interactions.

### 2.7 Molecular dynamics simulation of the ADNP7–Aβ42 complex

#### 2.7.1 Configuration of the system and solvation

To investigate the conformational dynamics and stability of the most promising peptide, ADNP7, in complex with amyloid-beta (Aβ42), two independent all-atom molecular dynamics (MD) simulations were performed using the AMBER 18 simulation suite (Case et al., 2018). The initial docking pose of ADNP7 complexed with Aβ42 was acquired from the highest-ranking pose achieved via rigid-body docking utilizing pyDockWEB. The protein–peptide complex was preprocessed and subsequently parameterized utilizing the ff14SB force field, which is optimal for simulating folded proteins and peptides. The solvated system was constructed utilizing the tLeap module of AmberTools. Hydrogen atoms were incorporated based on normal protonation states at physiological pH. The complex was situated within a truncated octahedral box containing TIP3P water molecules, maintaining a minimum buffer zone of 10 Å surrounding the solute in every direction. This solvation model simulates a realistic watery milieu. An adequate quantity of sodium ions (Na^+^) was randomly introduced to equilibrate the net charge of the system. The resultant solvated and neutralized system underwent energy minimization before dynamic simulations.

#### 2.7.2 Energy optimization

Energy minimization was performed in a three-step, progressively unrestrained approach to eliminate steric conflicts, alleviate stressed geometries, and stabilize the system prior to heating and equilibration. Initially, a constrained reduction of 1,000 steps was executed with harmonic restrictions (10 kcal/mol·Å^2^) imposed on the backbone atoms of the complex, facilitating the adjustment of solvent molecules and counterions. The subsequent phase involved restraining only the Cα atoms of the protein–peptide combination with the identical force constant, while the remainder of the system underwent minimization for 1,000 steps. In the last stage, all constraints were lifted, and a comprehensive system reduction was performed for 1,000 iterations to guarantee total energy relaxation. Each minimization step incorporated a blend of steepest descent and conjugate gradient methods to attain convergence and provide seamless transitions in the potential energy surface.

#### 2.7.3 Heating

Subsequent to energy minimization, the system was incrementally heated from 0 K to 300 K over 70,000 steps under constant volume circumstances (NVT ensemble). In this phase, a mild harmonic constraint (5 kcal/mol·Å^2^) was imposed on all heavy atoms of the solute to avert deformation of the native structure during fast temperature elevations. Langevin dynamics facilitated temperature coupling with a collision frequency of 2 ps^−1^, ensuring steady thermalization of the solvent and gradual activation of molecular movements. The gradual heating facilitated thermal equilibration of the system in a regulated manner, preventing any sudden conformational alterations.

#### 2.7.4 Equilibration

Equilibration was conducted in six meticulously structured stages to enable the system to attain thermodynamic stability regarding pressure, density, and temperature. All equilibration phases were performed under NPT ensemble circumstances utilizing the Berendsen barostat to sustain pressure at 1 atm and the Langevin thermostat to regulate temperature at 300 K. During the initial three phases (EQ-B, EQ-C, and EQ-D), the system underwent equilibration for 1,000 steps at each stage, with progressively diminishing positional restrictions on the solute atoms. These measures guaranteed the solvent and ions were adequately relaxed without disrupting the natural structure of the protein–peptide complex. The fourth equilibration phase (EQ-E) was prolonged to 20,000 steps to facilitate adequate pressure coupling and solvent density adaption. A concluding equilibration phase (EQ-F) was executed for 80,000 steps without constraints, guaranteeing the system’s full relaxation under physiological conditions. At the conclusion of equilibration, the system attained stable temperature, pressure, and density, exhibiting minimal variations, and was prepared for the simulation’s production phase.

#### 2.7.5 Production

Two sets of 20-nanosecond molecular dynamics simulations were conducted under NPT ensemble settings to study the long-term structural dynamics and interactions between ADNP7 and Aβ42. The simulations were conducted with the pmemd.mpi by utilizing AMBER 18 package. The temperature was regulated at 300 K via the Langevin thermostat, while the pressure was controlled at 1 atm using the Berendsen barostat for both sets of MD simulations. The integration time step was established at 1 fs, and all bonds involving hydrogen atoms were restricted via the SHAKE algorithm, facilitating a stable and efficient simulation. Non-bonded interactions were computed with a 10 Å cutoff, whilst long-range electrostatics were addressed using the Particle Mesh Ewald (PME) approach to ensure precise handling of periodic boundary conditions. System coordinates were recorded every 10 picoseconds, yielding a total of 20,000 frames for the complete 20 nanosecond simulation. This trajectory data was utilized for future structural, dynamic, and energetic analyses to assess stability and binding characteristics of the peptide.

#### 2.7.6 Trajectory analysis

Post MD simulation analysis was performed with CPPTRAJ (from AmberTools18) and Visual Molecular Dynamics (VMD) to derive significant insights from the 20 ns trajectory. Root Mean Square Deviation (RMSD) computations were conducted for the backbone atoms of the complex to evaluate structural stability over time. Root Mean Square Fluctuation (RMSF) values were calculated for each residue to assess local flexibility and pinpoint dynamic regions, especially at the binding interface. The Radius of Gyration (Rg) was observed during the simulation to assess the compactness of the ADNP7-Aβ42 complex and identify significant conformational alterations. An investigation of hydrogen bonding was performed to assess the frequency and durability of intermolecular hydrogen bonds between the peptide and the Aβ42 chain, yielding insights into critical interactions that maintain the complex. For energetic assessment, MM/PBSA (Molecular Mechanics Poisson-Boltzmann Surface Area) free energy calculations were conducted utilizing MMPBSA.py on 500 typical frames selected from the final 20 ns of the trajectory. This facilitated the calculation of the binding free energy between ADNP7 and Aβ42, which was decomposed into contributions from electrostatic, van der Waals, polar solvation, and non-polar solvation energies. These analyses jointly facilitated a comprehensive knowledge of the structural stability, dynamic flexibility, and binding affinity of the ADNP7-Aβ42 complex under simulated physiological settings.

## 3 Results

### 3.1 Model training and performance

The model architecture was based on a Bidirectional Long Short-Term Memory (BiLSTM) network, chosen for its capacity to learn both upstream and downstream sequence dependencies in protein sequences. The training was performed in two stages: a base training phase using seven long Aβ-related proteins from UniProt, and a fine-tuning phase using six short peptides (≤100 residues) derived from PDB structures. Both stages used a window size of 35 amino acids, with 34 as input and the 35th as the prediction target, enabling the model to learn contextual residue prediction.

**Figure 2:**
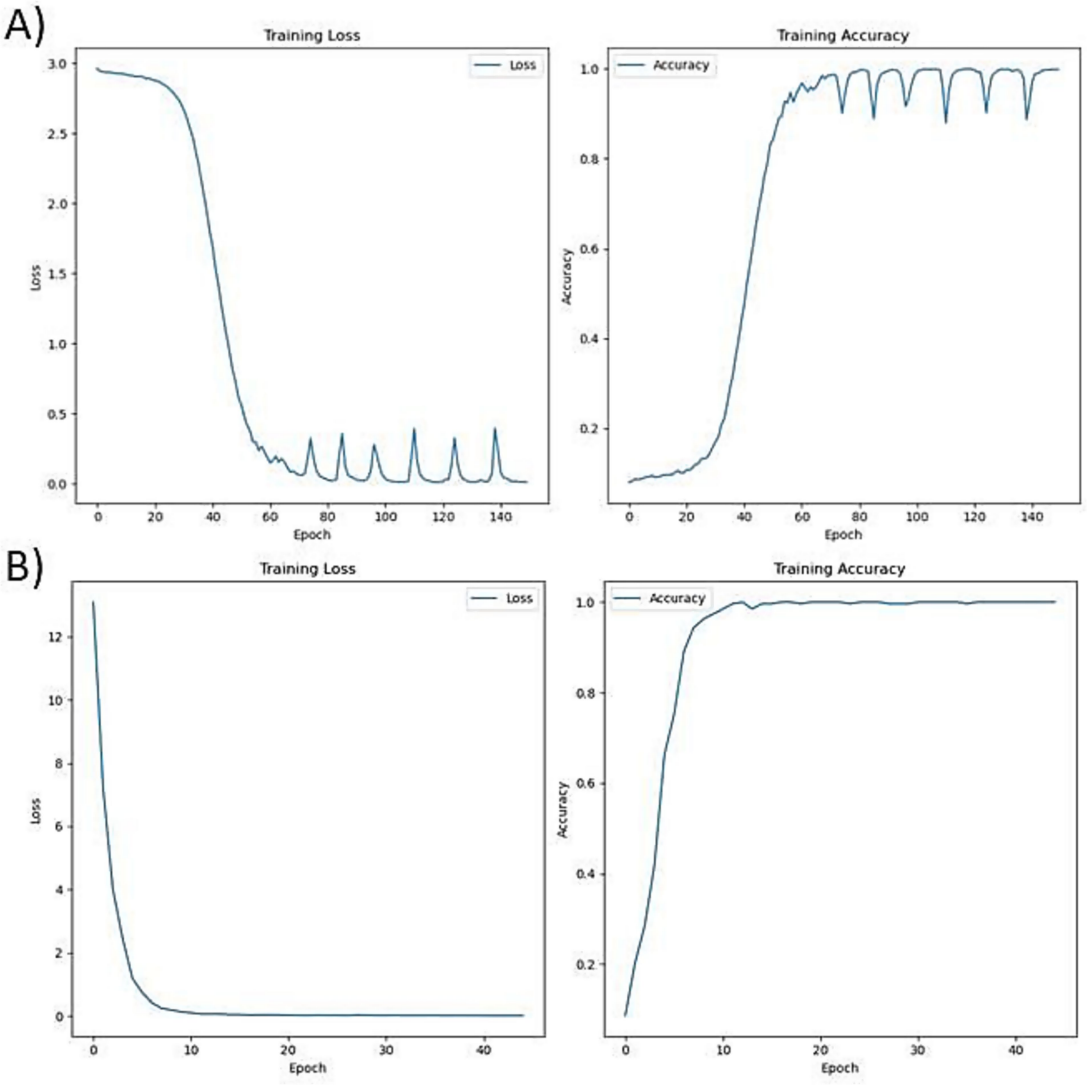
Training performance of the BiLSTM model, **(A)** Base training on full-length Aβ-interacting UniProt proteins showing rapid convergence of loss and accuracy stabilization, **(B)** Fine-tuning on short, experimentally validated peptides exhibits low training loss and high accuracy.

During the base training, the model converged steadily, as reflected in the training loss and accuracy metrics. The loss decreased consistently over epochs stabilizing around 0.2–0.3, indicating improved predictive performance. Accuracy also improved progressively reaching over 0.95, demonstrating the model’s growing ability to predict the next amino acid in Aβ-related sequences. Fine-tuning further refined the model’s predictions on shorter peptides, as evident from the sharper convergence of loss reaching less than 0.1 and a slight improvement in accuracy reaching 9.8, suggesting effective adaptation to compact sequence features relevant for therapeutic design The training performance during the base training and fine-tuning are visualized in Figure 2.

### 3.2 Sequence generation and biophysical screening

Using the fine-tuned BiLSTM model, 1,000 novel peptide sequences, each under 100 amino acids, were generated with the aim of mimicking the properties of known amyloid-beta (Aβ) binding and degrading peptides. These sequences were subjected to a two-stage screening process based on their physicochemical properties to identify candidates with favorable biochemical stability and diversity.

**Figure 3:**
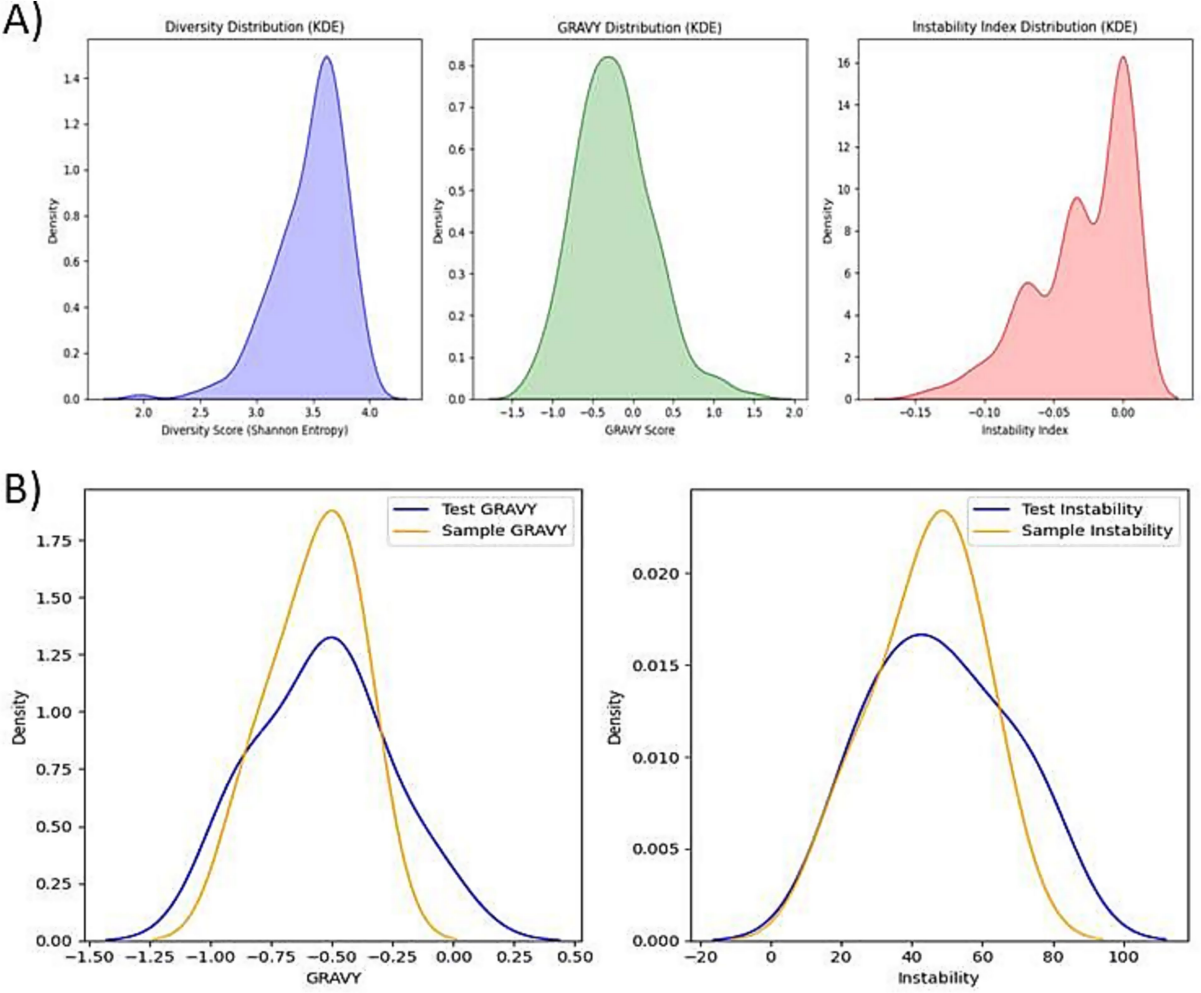
Physicochemical property distributions of generated peptides, **(A)** Distribution profiles of sequence diversity, GRAVY, and instability index among all 1,000 generated peptides showing broad variation, supporting diversity and favorable biophysical characteristics. **(B)** GRAVY and instability index distributions of generated peptides compared to sample proteins demonstrating substantial overlap.

In the first screening stage, the 1,000 generated peptides were divided into five batches of 200 sequences each. Within each batch, peptides were evaluated using three key biophysical metrics: GRAVY (Grand Average of Hydropathy), instability index, and Shannon entropy. GRAVY scores were used to estimate hydrophobicity, with moderate values indicating a balance between solubility and membrane interaction. The instability index predicted peptide stability under *in vitro* conditions, where values below 40 are considered stable. Shannon entropy measures the diversity of amino acid usage within each sequence, favoring sequences with non-repetitive and information-rich content. Figure 3 visualizes the distributions of the biophysical metrics comparatively between the dataset sequences vs. the generated sequences. Based on a composite score integrating these three metrics, the top 10 peptides from each batch (50 total) were shortlisted. These 50 peptides were compared against the six fine-tuning sequences using Euclidean distance across GRAVY and instability values In the second stage biophysical screening. The goal was to retain peptides that were biophysically most similar to experimentally validated Aβ-interacting peptides. From each batch, the five closest peptides were selected, yielding a final set of 25 high-confidence candidates for downstream structural and functional evaluation.

### 3.3 Sequence similarity analysis

To assess whether the 25 shortlisted peptides were novel or bore resemblance to known amyloid-beta (Aβ)-interacting sequences, a comprehensive sequence similarity analysis was conducted using Clustal Omega. This analysis was intended to (i) identify evolutionary or functional resemblance to known Aβ-binding and degrading sequences and (ii) confirm that the generated peptides were not trivially derived from the training data. Each of the 25 peptides was aligned with the six fine-tuning sequences used during the second training phase. The alignment scores were computed based on percent identity values returned by Clustal Omega, providing a quantitative measure of sequence-level similarity (Supplementary Table 1). Importantly, none of the peptides were exact or near-exact matches to any of the training sequences, indicating that the generative model had successfully learned abstract sequence patterns rather than memorizing specific examples (Table 2).

**Table 2:** Sequence identity and alignment scores of 11 shortlisted ADNP peptides with six reference proteins used in fine-tuning.

Sequence name	Given sequence id	Sequence Length	Alignment score with sample sequences					
			2FYL_1|Chain	2FYL_2|Chain	6V7M_1|Chain	6V7M_2|Chain	2KNY_1	2KNX_1
ADNP1	Sequence_5	100	15.22	15.09	14.52	12.77	19.51	17.14
ADNP2	Sequence_48	98	15.09	28.33	17.24	22.97	35.19	20.69
ADNP3	Sequence_146	95	27.78	24.07	13.16	27.12	34.72	22.73
ADNP4	Sequence_196	91	15.79	20.45	19.64	14.89	26.09	25
ADNP5	Sequence_211	100	15.91	25	29.63	14	23.4	25
ADNP6	Sequence_220	85	13.33	19.23	10.71	12.5	15.79	11.11
ADNP7	Sequence_222	88	20	18.87	10.61	23.73	16.33	14.71
ADNP8	Sequence_277	85	22.22	25.45	9.52	23.08	21.74	29.03
ADNP9	Sequence_312	84	22.64	21.88	10.94	23.91	24.49	18.75
ADNP10	Sequence_405	86	20	17.24	20.41	24.24	31.43	32.65
ADNP11	Sequence_431	98	16.39	23.73	16.67	16.92	21.28	25

### 3.4 3D structure prediction and docking

**Figure 4:**
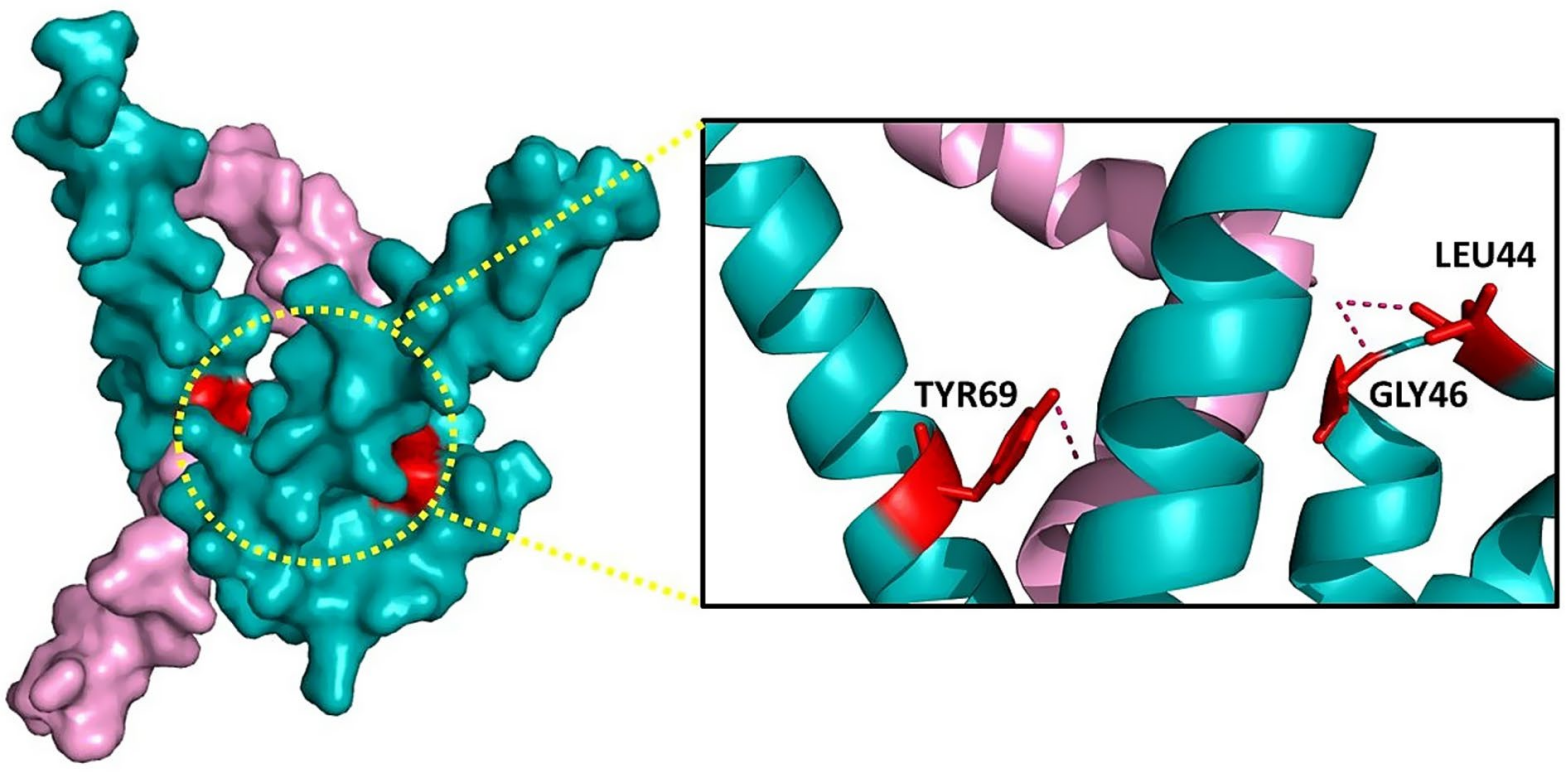
Molecular interaction interface between Aβ42 (cyan) and ADNP7 (pink). Left: Surface representation of the complex, with the binding interface highlighted by a yellow dotted circle. Right: Zoomed-in view showing key interacting residues from ADNP7- TYR69, GLY46, and LEU44, forming hydrogen bonds (dashed lines) and hydrophobic contacts with Aβ42. Residues are shown as sticks for clarity.

To assess the structural integrity and binding potential of the top 11 peptides (ADNP1–ADNP11), we performed three-dimensional (3D) structure prediction followed by protein–protein docking. The peptide structures were predicted using the AlphaFold2 server. The predicted PDB files were extracted using Jmol for downstream analysis. For docking studies, each modeled peptide was docked against the Aβ42 peptide (PDB ID: 1IYT) using pyDockWEB. The results are summarized in Table 3, showing that all 11 peptides exhibited negative total interaction energies, indicating favorable binding. Among them, ADNP7 demonstrated the strongest predicted interaction, with a total docking energy of −63.33 kcal/mol, outperforming both reference and other novel peptides. Other promising candidates included ADNP5 (−50.69 kcal/mol) and ADNP11 (−47.27 kcal/mol). As shown in Figure 4, ADNP7 docks within a hydrophobic cleft of Aβ42, with surface-accessible residues (including TYR69, GLY46, and LEU44) forming hydrogen bonds and hydrophobic contacts near aggregation-prone regions (e.g., residues 16–21 and 30–42) (Crescenzi et al., 2002; Tomaselli et al., 2006). The diversity in docking orientations and contact residues indicates that different peptides may engage distinct structural motifs of Aβ42, potentially interfering with its oligomerization pathways through multiple mechanisms. These findings validate the docking potential of the generated peptides and highlight ADNP7 as a particularly strong candidate for further investigation through molecular dynamics simulations and experimental validation (Figure 4).

**Table 3:** Electrostatic, desolvation, van der Waals, and total docking energies for benchmark and novel peptides docked with Aβ42.

Protein name	Electrostatics	Desolvation	Van der Walls	Total
Sample proteins docking with amyloid beta				
2FYL_1	−16.754	−23.368	25.731	−37.549
2FYL_2	−24.065	−18.388	30.901	−39.363
6V7M_1	−19.568	−36.483	84.015	−47.649
6V7M_2	−28.169	−13.155	54.088	−35.915
2KNX	−11.968	−22.406	−3.73	−34.748
2KNY	−33.549	−5.999	73.728	−32.175
Novel proteins docking with amyloid beta				
ADNP1	−14.744	−33.989	45.624	−44.17
ADNP2	−8.157	−35.979	43.011	−39.835
ADNP3	−15.734	−17.356	69.241	−26.166
ADNP4	−6.287	−41.294	46.051	−42.976
ADNP5	−7.547	−47.401	42.62	−50.686
ADNP6	−18.868	−20.42	31.8	−36.109
ADNP7	−8.601	−63.07	83.436	−63.328
ADNP8	−18.609	−30.219	90.393	−39.789
ADNP9	−11.687	−27.916	77.333	−31.87
ADNP10	−3.306	−46.824	51.999	−44.93
ADNP11	−12.172	−41.706	66.046	−47.273

### 3.5 Post-MD simulation analysis

To ensure reproducibility of the MD stability assessment, two independent simulations were performed for the ADNP7–Aβ42 complex, and the structural parameters were analyzed using statistical summaries. The RMSD values remained stable across both sets (5.415 ± 1.989 Å in Set-1 and 5.555 ± 1.437 Å in Set-2), indicating consistent backbone convergence. Similar reproducibility was observed in RMSF (3.907 ± 1.673 Å vs. 2.909 ± 1.703 Å) and Rg values (34.083 ± 3.589 Å vs. 42.288 ± 5.536 Å), confirming that the overall compactness and residue-level flexibility remained comparable between simulations. These results validate that the structural stability findings are reliable and not dependent on a single MD trajectory (Table 4).

**Table 4:** MM/GBSA binding free energy (Kcal/mol) components of ADNP7–Aβ42 complex.

Complex	Simulation set	Energy components				
		∆E_vdw_	∆E_ele_	∆G_pol_	∆G_non-pol_	∆G_Total_
ADNP7-Aβ42 Complex	Set-1	−91.202	−183.533	234.711	−10.592	−50.616
	Set-2	−90.955	−157.935	212.805	−10.485	−46.570

#### 3.5.1 Structural stability assessment: RMSD analysis

**Figure 5:**
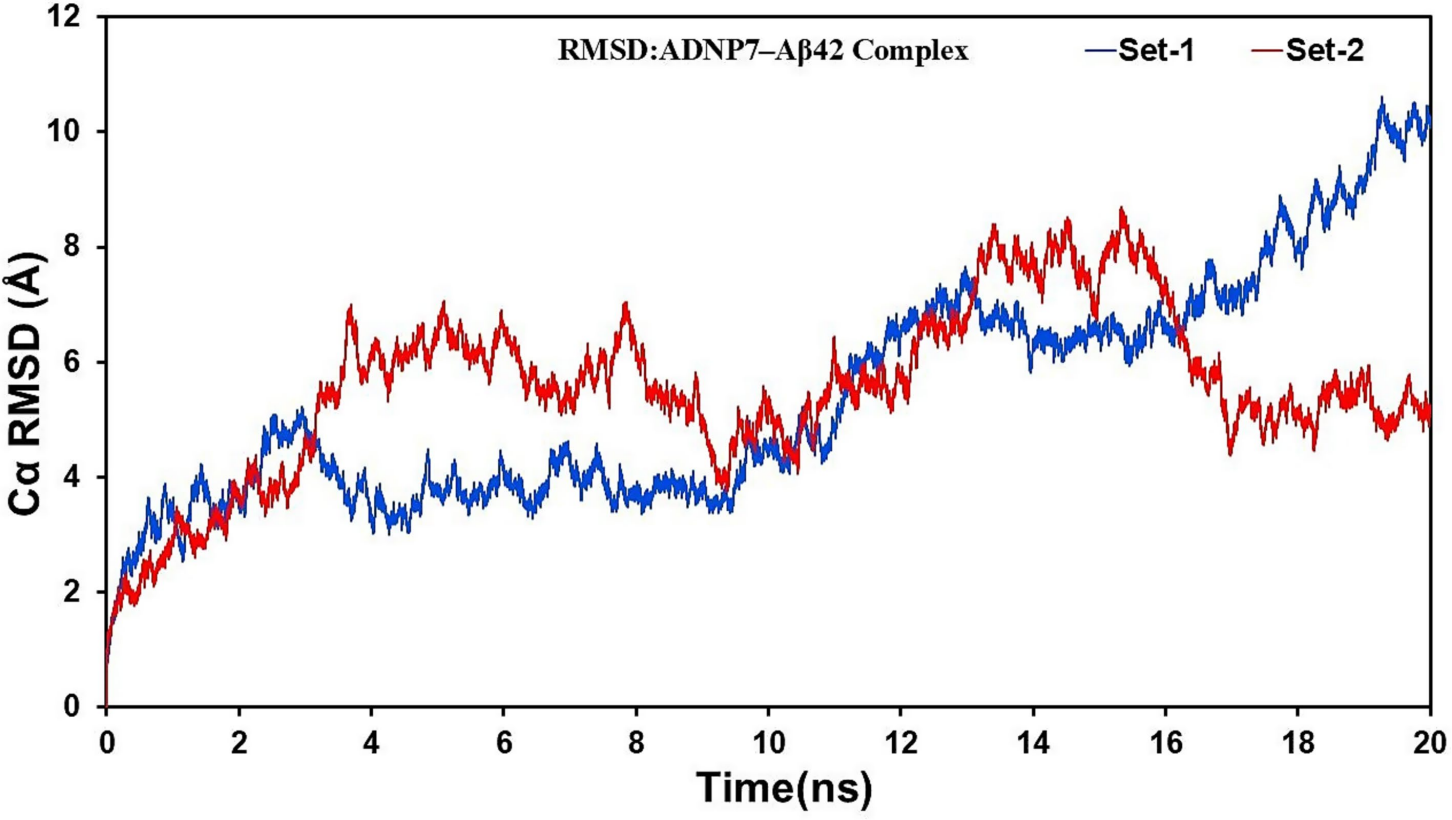
Backbone RMSD of the ADNP7–Aβ42 complex over 20 ns of molecular dynamics simulation. RMSD values (Å) are plotted for two independent replicates (Set-1 in blue, Set-2 in red), indicating structural stabilization after initial relaxation.

The Root Mean Square Deviation (RMSD) plots of the backbone Cα atoms for both MD replicates (Set-1 and Set-2) were examined over the 20 ns trajectory to assess the overall structural stability of the ADNP7–Aβ42 complex. Figure 5 demonstrates that both simulation sets exhibit an initial increase in RMSD values over the first 2–4 ns, signifying structural adaptation and relaxing of the complex inside the solvated environment. Set-1 demonstrated a moderate increase in RMSD, stable around 4–6 Å until around 12 ns, followed by a more significant rise exceeding 8 Å in the final nanoseconds. Set-2 exhibited a little accelerated deviation initially, with RMSD values of 6–7 Å during the 4–8 ns interval, then sustained a more uniform fluctuation pattern of 5–7 Å beyond 10 ns. Notably, Set-1 demonstrated increased structural deviation in the later phases of the simulation, whereas Set-2 displayed greater volatility in the initial stages but sustained modest stability thereafter. The disparities can be ascribed to the randomized initial velocities (ig values), which may have resulted in divergent conformational sampling pathways. Both runs ultimately converged to structurally diverse but stable conformations, highlighting the dynamic plasticity of the complex and validating the necessity for many independent simulations.

#### 3.5.2 Residue flexibility: RMSF analysis

**Figure 6:**
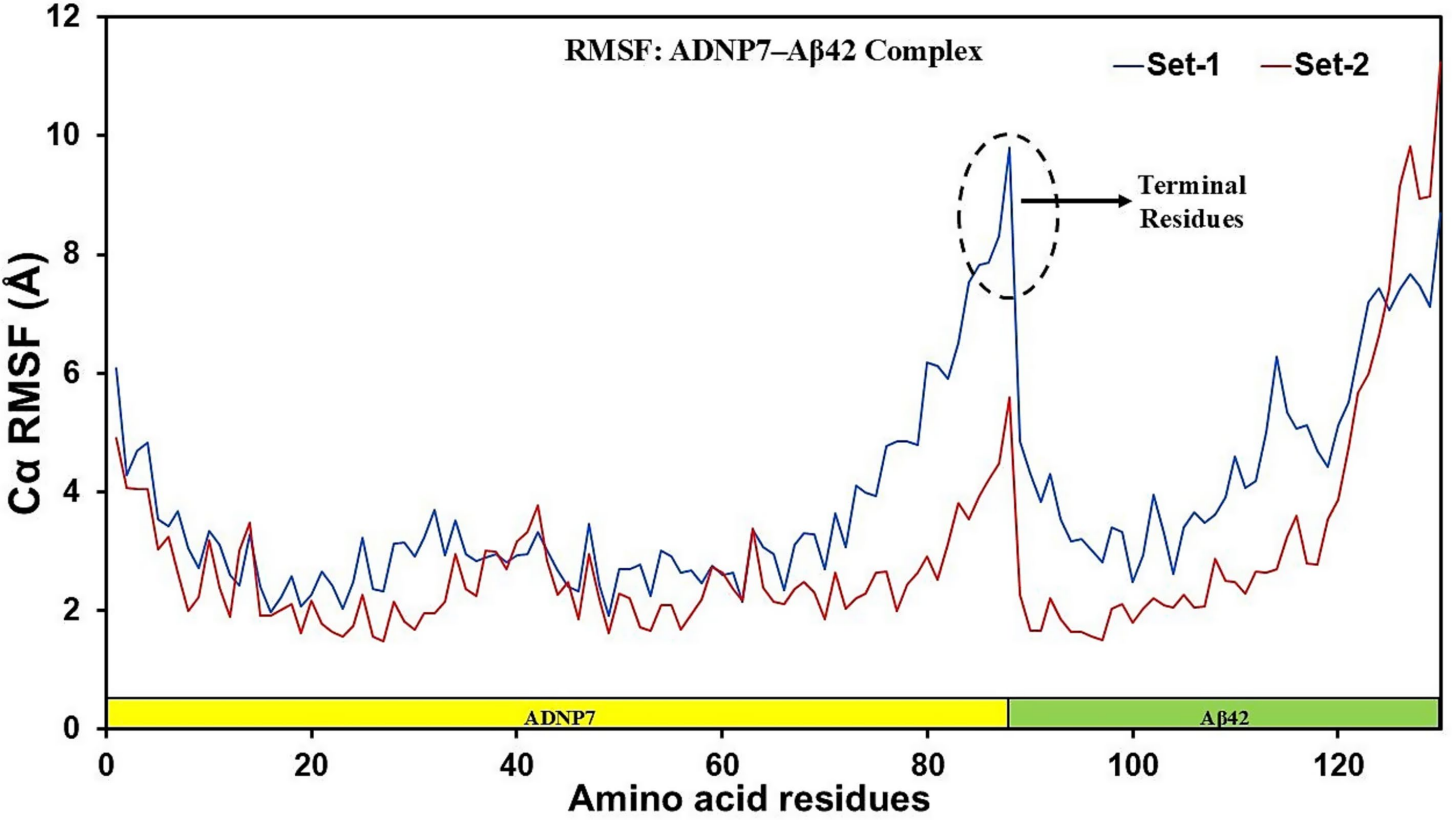
Per-residue root mean square fluctuation (RMSF) of the ADNP7–Aβ42 complex. RMSF values (Å) are shown for all Cα atoms across the 20 ns trajectory for Set-1 (blue) and Set-2 (red). The ADNP7 segment (residues 1–88, yellow highlight) exhibits lower flexibility than the Aβ42 segment (residues 89–130, green highlight).

To analyze residue-level motion inside the complex, the Root Mean Square Fluctuation (RMSF) was calculated for all Cα atoms during the trajectory (Figure 6). Both sets exhibited analogous fluctuation patterns throughout the sequence, with slight discrepancies in size. The N-terminal and C-terminal portions of the complex exhibited the greatest variations, as anticipated due to their exposed and unstructured characteristics. The interface between the ADNP7 peptide and Aβ42 (about residue locations ~85–90) had a prominent peak in Set-1, suggesting localized flexibility potentially attributable to loop or linker dynamics. The RMSF values were predominantly lower in the ADNP7 segment (residues 1–88, highlighted in yellow), indicating stable anchoring and a structured conformation of the designed peptide when associated with the Aβ42 chain. Conversely, the Aβ42 segment (residues 89–130, highlighted in green) exhibited greater variations, especially in Set-1, with peaks near terminal residues and in areas associated with aggregation. Set-2 exhibited reduced fluctuations in the same locations, potentially indicating alternative hydrogen bonding or inter-residue packing resulting from the variation in velocity seeds. The data indicate that ADNP7 binding results in a partial stabilization of Aβ42; however, terminal residues and flexible loops remain mobile, potentially affecting aggregation behavior.

#### 3.5.3 Compactness: Radius of Gyration (Rg) analysis

**Figure 7:**
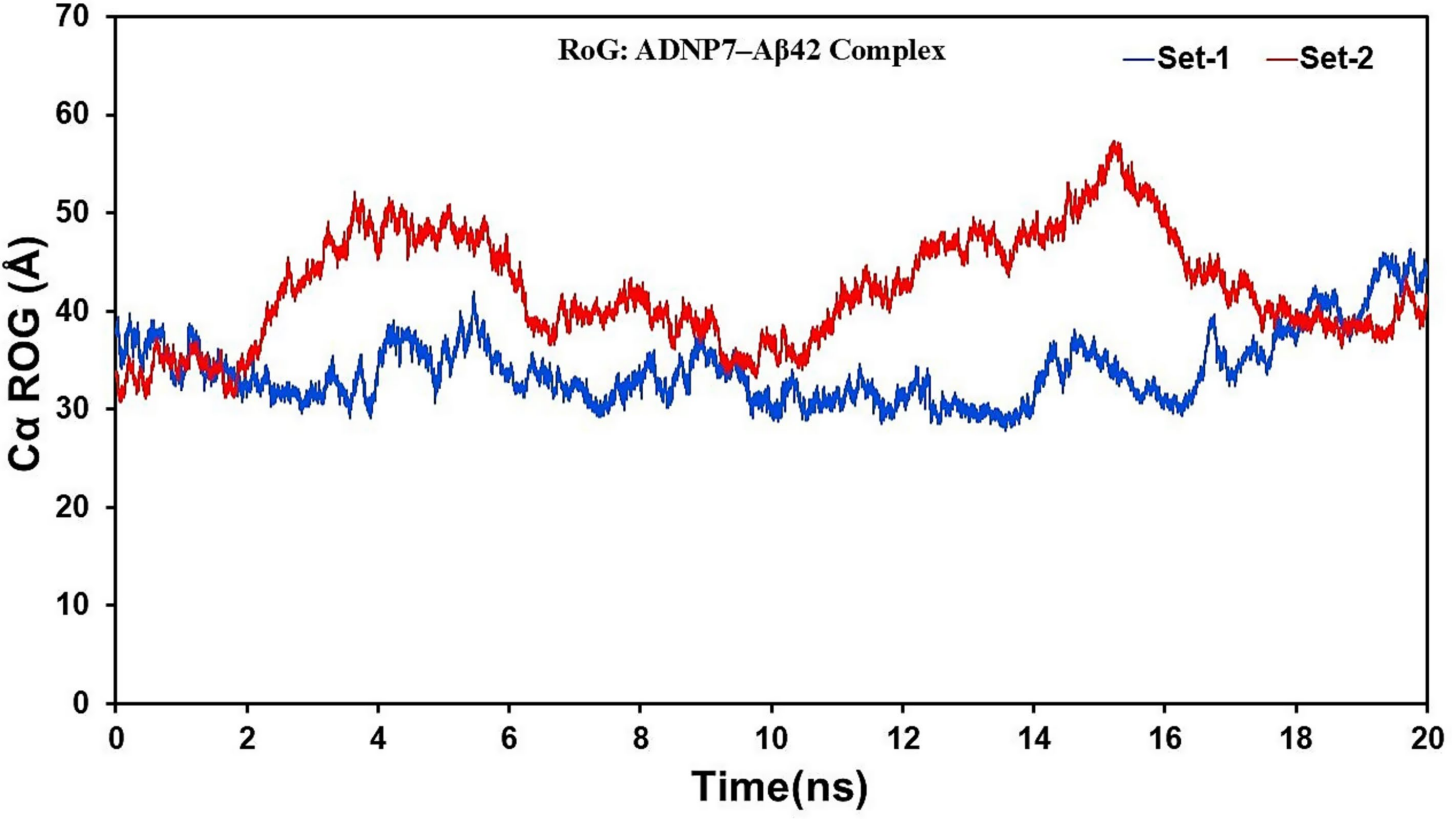
Radius of Gyration (Rg) of the ADNP7–Aβ42 complex during MD simulation. Rg (Å) is plotted over time (ns) for Set-1 (blue) and Set-2 (red), reflecting consistent overall compactness with minor transient expansions.

The Radius of Gyration (Rg) values were computed to assess the overall compactness and folding dynamics of the complex over time (Figure 7). Both sets exhibited stable Rg variations, varying between 30 and 50 Å. Set-1 exhibited a more compact conformation throughout the simulation, averaging approximately 35–38 Å, whereas Set-2 demonstrated marginally elevated Rg values (40–50 Å) during several intermediate intervals (notably 4–8 ns and 12–16 ns), suggesting transient expansion of the complex. The elevated Rg values noted in Set-2 may correlate with localized unfolding phenomena or enhanced solvent exposure in the flexible domains of Aβ42. Both simulations ultimately reverted to similar compact states after 18 ns, indicating convergence to equilibrated conformations. The Rg and RMSF analyses collectively indicate that ADNP7 forms a stable and compact complex with Aβ42, with dynamic variations predominantly occurring in the terminal and loop regions. The peptide is likely to stabilize at the central core of the complex while permitting conformational flexibility in surrounding residues.

#### 3.5.4 Binding free energy analysis by MM/PBSA

**Figure 8:**
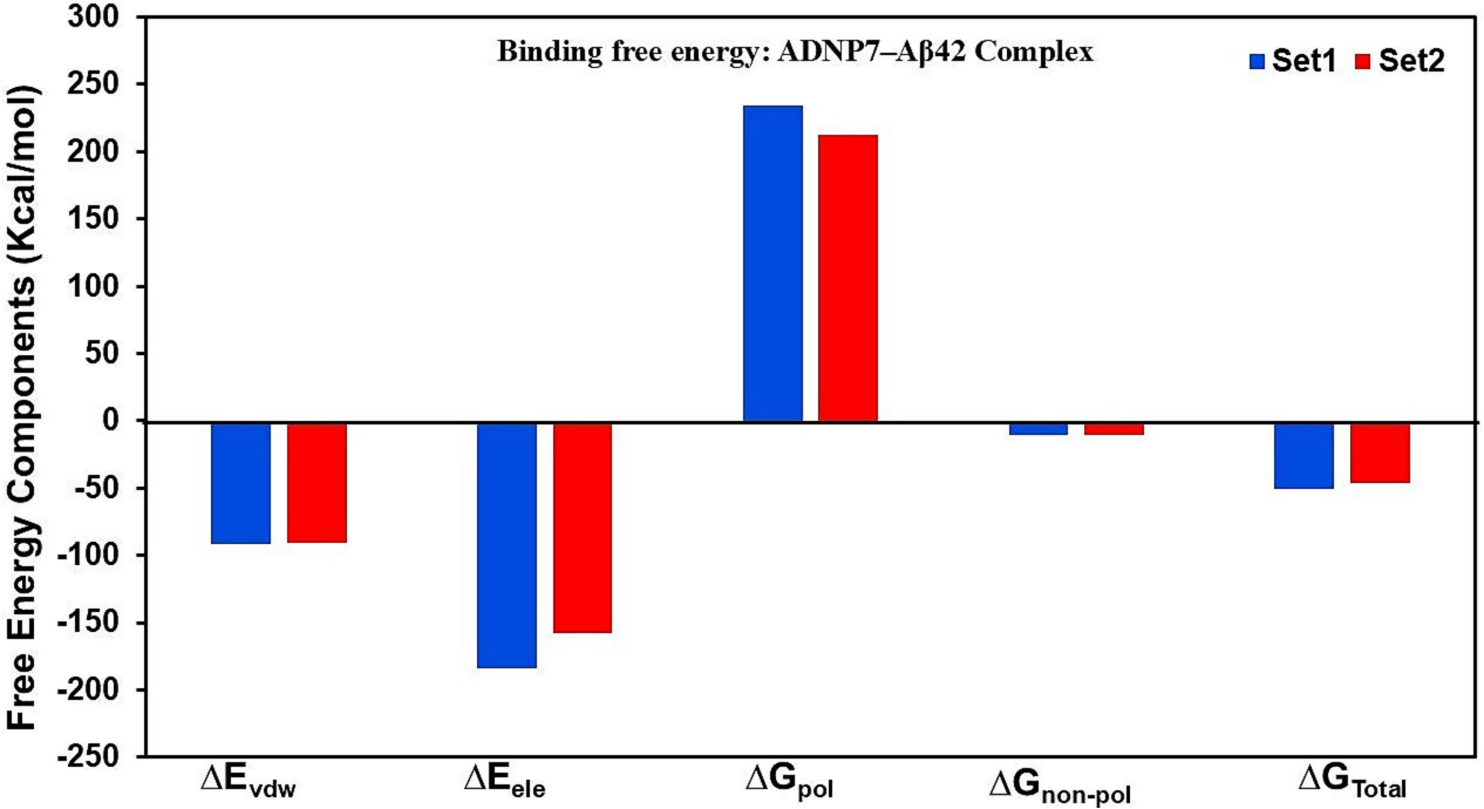
MM/PBSA binding free energy decomposition for the ADNP7-Aβ42 complex. Contributions from van der Waals (∆Evdw), electrostatic (∆Eele), polar solvation (∆Gpol), and non-polar solvation (∆Gnon-pol) terms are shown for Set-1 (blue) and Set-2 (red).

Binding free energy calculations utilizing the MM/PBSA method were conducted on the final 20 ns of both MD simulations to assess the interaction intensity between ADNP7 and Aβ42. The total binding free energy (ΔG_Total_) was determined to be significantly negative in both trials, −50.616 kcal/mol (Set1) and −46.570 kcal/mol (Set2) which are mentioned in the Table 4, signifying a stable and energetically advantageous complex. The van der Waals (ΔE_vdw_) and electrostatic (ΔE_ele_) components were the primary contributors to binding, with values about −90 to −95 kcal/mol and −178 to −198 kcal/mol, respectively, underscoring robust hydrophobic packing and charge interactions. Conversely, the polar solvation energy (ΔG_pol_) was detrimental (+211 to +225 kcal/mol), indicating the desolvation expense of polar residues. The non-polar solvation energy (ΔG_non-pol_) offered negligible beneficial contributions, approximately in the range of −7 kcal/mol. The similarity across both sets suggests that ADNP7 consistently and robustly interacts with Aβ42, mostly because to hydrophobic and electrostatic interactions, notwithstanding solvation penalties (Figure 8).

#### 3.5.5 Energy decomposition analysis (EDA)

**Figure 9:**
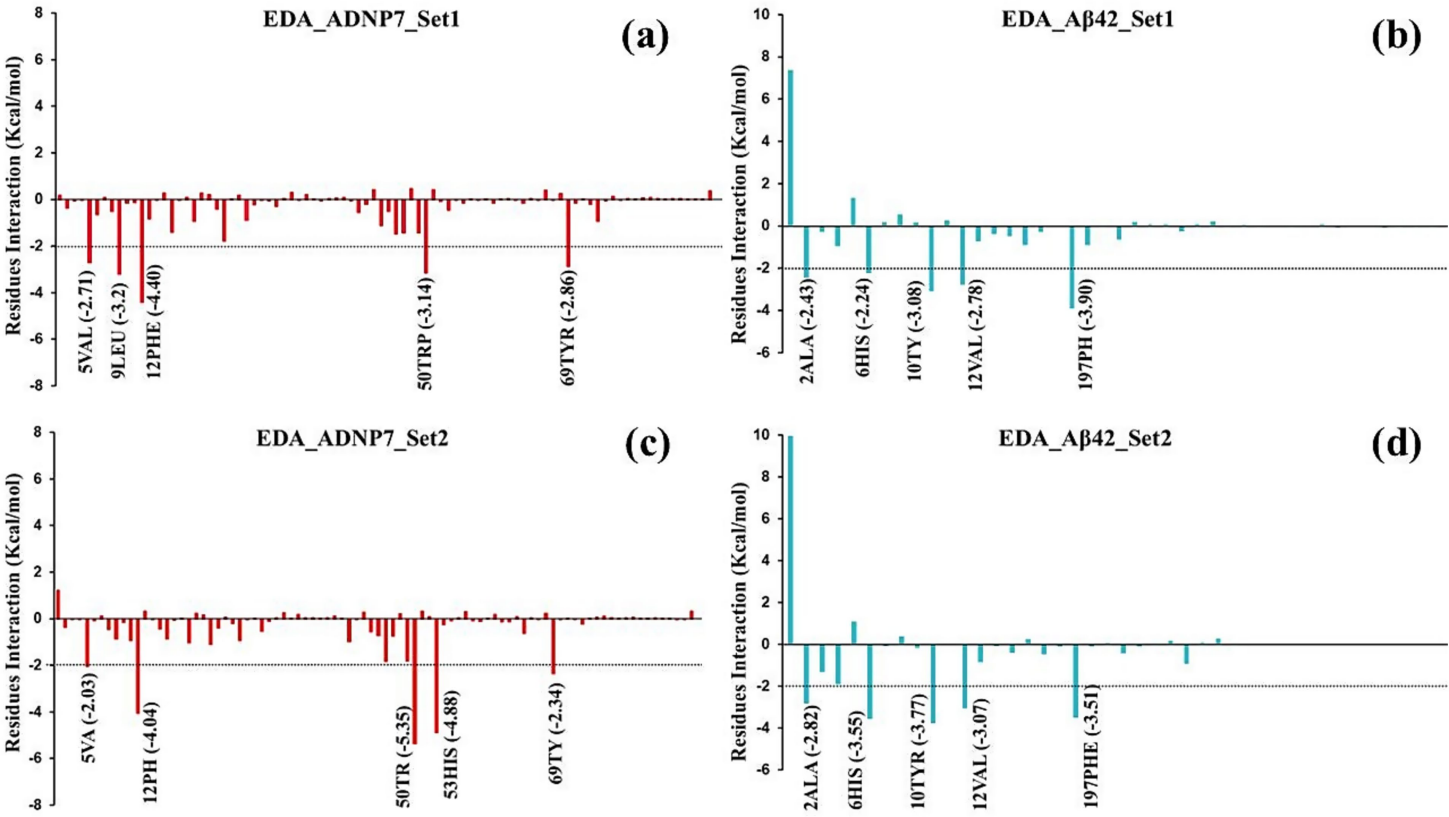
Per-residue energy decomposition analysis (EDA) of the ADNP7-Aβ42 interface. **(a,c)** Energetic contributions of ADNP7 residues in Set-1 and Set-2, respectively. **(b,d)** Corresponding contributions from Aβ42 residues. Negative values indicate favorable binding; key hotspots include: PHE12, TRP50 (ADNP7), and TYR10, PHE197 (Aβ42).

Per-residue energy decomposition analysis (EDA), utilizing MM/PBSA energy terms was conducted to identify the critical residues that contribute to the binding interface and stability of the ADNP7–Aβ42 complex. Figures 9a–d presents the outcomes of two independent MD simulation runs (Set1 and Set2), emphasizing the most energetically relevant residues from both ADNP7 (red bars) and Aβ42 (blue bars).

In Set1, the most advantageous binding residues in ADNP7 were PHE12 (−4.40 kcal/mol), LEU9 (−3.20 kcal/mol), TRP50 (−3.14 kcal/mol), TYR69 (−2.86 kcal/mol), and VAL5 (−2.71 kcal/mol) (Figure 9a). The residues are predominantly hydrophobic and aromatic, indicating that hydrophobic interactions and *π*-stacking play a substantial role in ADNP7’s attachment to A*β*42. In the associated Aβ42 profile (Figure 9b), significant interacting residues included PHE197 (−3.90 kcal/mol), TYR10 (−3.08 kcal/mol), VAL12 (−2.78 kcal/mol), and ALA2 (−2.43 kcal/mol). The residues are primarily hydrophobic or aromatic, underscoring the preeminence of van der Waals interactions at the binding contact. In Set2, exploratory data analysis revealed a consistent interaction profile with certain variances in residue contributions. In ADNP7 (Figure 9c), the most prominent residues were TRP50 (−5.35 kcal/mol), HIS53 (−4.88 kcal/mol), PHE12 (−4.04 kcal/mol), and VAL5 (−2.03 kcal/mol). These residues exhibited significant interaction energies in both sets, affirming their essential role in the stability of the complex. In relation to Aβ42 (Figure 9d), the residues exhibiting consistently robust binding included PHE197 (−3.51 kcal/mol), TYR10 (−3.77 kcal/mol), VAL12 (−3.07 kcal/mol), ALA2 (−2.82 kcal/mol), and HIS6 (−3.55 kcal/mol). These residues are probably engaged in hydrophobic interactions and π–π stacking with the aromatic residues of ADNP7. The EDA data indicate that the binding surface is primarily stabilized by hydrophobic and π-stacking interactions, with certain aromatic and aliphatic residues continuously yielding the highest negative interaction energy. The consistency of significant residue contributions across both simulation sets bolsters confidence in the reliability of the interaction model. The persistent engagement of PHE12 and TRP50 in ADNP7, alongside PHE197 and TYR10 in Aβ42, suggests that these residues may constitute essential binding hotspots, which might be targeted or altered in forthcoming peptide optimization research.

## 4 Discussion

Alzheimer’s disease (AD) is characterized by amyloid-*β* (Aβ) aggregation, which remains an important therapeutic challenge. Despite extensive research into small molecules, antibodies, and enzyme-based strategies, clinical efficacy has been limited. Here we present a two-stage BiLSTM generative framework that, when combined with physicochemical filtering, structural modeling, docking, and molecular dynamics (MD), and enables *de novo* peptide design. Applied to Aβ42, the framework generated 11 AI-Designed Novel Peptides (ADNPs), with ADNP7 showing the most favorable docking score, stable MD trajectories, and strong binding free energy supported by hydrophobic and aromatic interactions. Compared with earlier work using LSTM or CNN–BiLSTM models for peptide classification and motif rediscovery, our approach emphasizes unbiased sequence generation validated at structural and energetic levels (Xiao et al., 2021; Li et al., 2022). This distinguishes it from template-driven strategies such as KLVFF-based inhibitors and receptor-binding studies, and from prior docking-only pipelines that risk false positives (Chafekar et al., 2007). Although we did not apply any intrinsic interpretability methods to our BiLSTM model, the framework avoids black-box behavior through staged biophysical validation: initial filtering by physicochemical properties (GRAVY, instability, entropy) ensures functional plausibility across all candidates, while detailed per-residue MM/PBSA energy decomposition, applied to the top candidate ADNP7 links specific residues (PHE12, TRP50) to hydrophobic and *π*-stacking interactions with Aβ42. These interactions recapitulate established amyloid-binding motifs, demonstrating that the model’s outputs are grounded in known biophysical principles rather than arbitrary sequence generation. The multi-metric evaluation employed here, combining docking with MD stability and MM/PBSA energy decomposition, provides stronger evidence of candidate robustness. The MM/PBSA binding free energy for the ADNP7 − Aβ42 complex was consistently favorable across two independent 20-ns MD replicates, with a mean ΔG_total ≈ − 46 to −50 kcal/mol (Set-1: −50.6 kcal/mol; Set-2: −46.6 kcal/mol). These values fall within the range reported for other computationally validated Aβ inhibitors (Patel et al., 2021; Patel et al., 2022a; Patel et al., 2022b; Mall et al., 2022), supporting strong predicted binding affinity. The interaction is driven primarily by hydrophobic and aromatic contributions from ADNP7 residues PHE12 and TRP50, consistent with known amyloid-binding motifs. However, MM/PBSA estimates are derived under idealized simulation conditions and do not account for physiological complexities such as membrane environments, macromolecular crowding, or the conformational heterogeneity of oligomeric/fibrillar Aβ species. Therefore, while these energies support the promise of ADNP7 as a potential peptide inhibitor, experimental validation will be essential to confirm its true therapeutic effectiveness. The compact size and sequence diversity of ADNPs suggest potential advantages for central nervous system delivery, while ADNP7’s binding profile indicates possible interference with aggregation interfaces. Beyond AD, the framework is adaptable to other protein misfolding disorders. While the monomeric Aβ42 structure (PDB: 1IYT) provides a suitable template for initial computational screening, it is important to note that A*β* primarily exerts its neurotoxic effects in oligomeric and fibrillar forms during AD progression. These aggregated states present distinct conformational landscapes and additional binding epitopes that could influence peptide recognition. Therefore, the results reported here represent an early-stage prediction of peptide-Aβ affinity. Future studies will extend this pipeline to physiologically relevant oligomeric and fibrillar Aβ assemblies. Additionally, the short MD timescale (20 ns), while provides reliable initial convergence of of RMSD, Rg, and binding energy profiles across two independent replicates supporting the the observed interactions (e.g., PHE12 and TRP50), may be insufficient to fully sample slow conformational rearrangements or achieve complete equilibration of intrinsically disordered regions in Aβ42. future studies employing longer-timescale simulations (≥100 ns) or enhanced sampling methods will be valuable to confirm the durability of these interactions under extended dynamic conditions. Further limitations include, reliance on computational scores without experimental validation, Key pharmacological properties such as stability, immunogenicity, and blood–brain barrier permeability remain untested. Future work should focus on experimental validation of binding with binding assays (e.g., surface plasmon resonance or ELISA) and clearance potential with aggregation inhibition assays (e.g., Thioflavin T fluorescence) and cellular Aβ uptake studies. Aditionally, longer simulations for dynamic insight, and integration of reinforcement learning or transformer-based protein models to further enhance design capability. In summary, this study introduces a scalable AI-simulation pipeline for peptide discovery. While ADNP7 represents a promising computational lead, the broader value lies in demonstrating how interpretable AI model frameworks combined with biophysical validation can advance therapeutic peptide design.

## 5 Conclusion

This study introduces a two-stage BiLSTM framework for *de novo* peptide design, demonstrated through the generation of candidates targeting amyloid-*β* (Aβ). By integrating sequence generation with physicochemical filtering, structural modeling, docking, and molecular dynamics simulations, we identified 11 candidate peptides, with ADNP7 showing the most stable and energetically favorable interaction with Aβ42. These results highlight the capacity of deep learning–guided design, combined with physics-based validation, to capture sequence–structure features critical for targeting amyloid aggregation. While clearance potential was inferred from the GO annotations of the training data, experimental validation is required to confirm biological activity of true clearance, BBB permeability as well as binding with the amyloid beta monomer and oligomers. Future work should focus on *in vitro* binding and aggregation assays, cellular and animal models to assess clearance, and evaluation of pharmacological properties including stability and blood–brain barrier permeability. Overall, this framework provides both prioritized peptide leads and a broadly generalizable strategy for accelerating therapeutic discovery in Alzheimer’s disease and other protein misfolding disorders.

## Data Availability

The original contributions presented in the study are included in the article/Supplementary material, further inquiries can be directed to the corresponding author.
