# Supplementary material for: Designing novel peptides with amyloid-β binding and clearance potential using BiLSTM and molecular dynamics
Source: Front Artif Intell. 2025 Nov 19;8:1709505. doi: 10.3389/frai.2025.1709505 (PMC12673400; doi:10.3389/frai.2025.1709505)
Supplement: Supplementary file 1 [file Table_1.docx]

Designing Novel Peptides with Amyloid-β Binding and Clearance Potential Using BiLSTM and Molecular Dynamics

Vinod Kumar Yata^1^, Om Pritam Das^2^, Jarmani Dansana^3^, Abhishikta Gadtya^3^, Biswa Ranjan Meher^3^, Sarad Pawar Naik Bukke^4^, Narasaiah Kolliputi^5^*

^1^Department of Biotechnology, School of Allied and Healthcare Sciences, Malla Reddy University, Hyderabad, Telangana, 500100, INDIA

^2^Department of Molecular Biology, Central University of Andhra Pradesh, Ananthapuramu, Andhra Pradesh 515701, INDIA

^3^Computational Biology and Bioinformatics Laboratory, PG Department of Botany, Berhampur University, Berhampur, Odisha-760007, INDIA

^4^Department of Pharmaceutics and Pharmaceutical Technology, Kampala International University, Western Campus, P.O. Box 71, Ishaka - Bushenyi, Uganda

^5^Division of Allergy and Immunology, Department of Internal Medicine, USF Morsani College of Medicine, Tampa, FL, USA.

*Corresponding author: Narasaiah Kolliputi, PhD

Division of Allergy and Immunology, Department of Internal Medicine, USF Morsani College of Medicine, Tampa, FL, USA.

[nkollipu@usf.edu](mailto:nkollipu@usf.edu)

Supplementary Table 1: Multiple sequence alignment results, showing the percent similarity between the generated proteins and the sample proteins.

| **Sl. no.** | **Generated Sequence id** | **2FYL_1 Chain** | **2FYL_2 Chain** | **6V7M_1 Chain** | **6V7M_2 Chain** | **2KNY_1 Chain** | **2KNX_1 Chain** |
| --- | --- | --- | --- | --- | --- | --- | --- |
| 1 | Sequence_77 | 22.95 | 7.5 | 16.07 | 23.64 | 21.95 | 7.69 |
| 2 | Sequence_48 | 15.09 | 28.33 | 17.24 | 22.97 | 35.19 | 20.69 |
| 3 | Sequence_43 | 7.69 | 38.24 | 10.17 | 11.76 | 7.84 | 5.56 |
| 4 | Sequence_6 | 0 | 16.28 | 2.27 | 5.71 | 17.78 | 18.6 |
| 5 | Sequence_146 | 27.78 | 24.07 | 13.16 | 27.12 | 34.72 | 22.73 |
| 6 | Sequence_56 | 0 | 16.67 | 15 | 8.33 | 3.57 | 0 |
| 7 | Sequence_24 | 25.93 | 0 | 13.56 | 24.24 | 23.53 | 0 |
| 8 | Sequence_117 | 4.76 | 44.12 | 13.73 | 9.38 | 12.5 | 0 |
| 9 | Sequence_155 | 20 | 33.33 | 23.4 | 28.57 | 19.23 | 0 |
| 10 | Sequence_15 | 0 | 30.77 | 23.08 | 22.95 | 27.59 | 28.57 |
| 11 | Sequence_220 | 13.33 | 19.23 | 10.71 | 12.5 | 15.79 | 11.11 |
| 12 | Sequence_318 | 8.7 | 14.06 | 6.9 | 8.11 | 16.67 | 17.65 |
| 13 | Sequence_211 | 15.91 | 25 | 29.63 | 14 | 23.4 | 25 |
| 14 | Sequence_222 | 20 | 18.87 | 10.61 | 23.73 | 16.33 | 14.71 |
| 15 | Sequence_215 | 18.92 | 38.24 | 18.52 | 18.18 | 17.24 | 0 |
| 16 | Sequence_174 | 15.38 | 6.98 | 10.94 | 11.11 | 14.81 | 30 |
| 17 | Sequence_5 | 15.22 | 15.09 | 14.52 | 12.77 | 19.51 | 17.14 |
| 18 | Sequence_35 | 8 | 16.13 | 14.58 | 3.7 | 8.33 | 11.11 |
| 19 | Sequence_82 | 15.22 | 18.52 | 24.56 | 8.57 | 13.16 | 13.89 |
| 20 | Sequence_196 | 15.79 | 20.45 | 19.64 | 14.89 | 26.09 | 25 |
| 21 | Sequence_431 | 16.39 | 23.73 | 16.67 | 16.92 | 21.28 | 25 |
| 22 | Sequence_435 | 11.36 | 7.14 | 24.49 | 7.32 | 8 | 0 |
| 23 | Sequence_312 | 22.64 | 21.88 | 10.94 | 23.91 | 24.49 | 18.75 |
| 24 | Sequence_277 | 22.22 | 25.45 | 9.52 | 23.08 | 21.74 | 29.03 |
| 25 | Sequence_405 | 20 | 17.24 | 20.41 | 24.24 | 31.43 | 32.65 |
